# Cross-cultural challenges in assessing medical professionalism among emergency physicians in a Middle Eastern Country (Bahrain): feasibility and psychometric properties of multisource feedback

**DOI:** 10.1186/s12245-016-0099-2

**Published:** 2016-02-03

**Authors:** Ahmed Al Ansari, Ahmed Al Meer, Mooza Althawadi, Deyari Henari, Khalid Al Khalifa

**Affiliations:** Department of General Surgery, Bahrain Defense Force Hospital, Off Waly Alahed Avenue, P. O. Box - 28743, West Riffa, Kingdom of Bahrain; Arabian Gulf University, Manama, Bahrain; Medical Education Department in the Royal College of Surgeons of Ireland in Bahrain, Adilya, Bahrain; Bahrain Defense Force Hospital, P. O. Box - 28743, Riffa, Kingdom of Bahrain; Department of Radiology, Bahrain Defense Force Hospital, P. O. Box - 28743, Riffa, Kingdom of Bahrain

**Keywords:** MSF system, Emergency, Validity, Generalizability

## Abstract

**Background:**

Multisource feedback (MSF) is an evaluation tool whereby surveys assessing physicians are administered among medical peers and colleagues. Such evaluations provide physicians with non-biased valuations of both their strengths and their weaknesses, offering an opportunity for improvement in their work. Studies have shown that MSF is particularly effective for emergency care physicians.

**Methods:**

The study was undertaken in a military teaching hospital in Bahrain. A total of 30 emergency physicians (the total number of emergency physicians in our hospital), 16 males and 14 females, were evaluated using multisource feedback. Each emergency physician was assessed by three groups of raters, including 4 emergency physicians, 4 referral physicians from other departments, and 4 coworkers from within the emergency department. Feasibility of the questionnaire was analyzed via response rates, average time required to complete it, and the number of raters required to produce reliable results. We used exploratory factor analysis to examine for the construct validity. Cronbach’s coefficient was calculated to measure the internal consistency reliability of the instrument.

**Results:**

The total mean response rate was 74.2 %, and the self-reported average time needed to fill out each survey was 4.3 min, indicating a good feasibility of the questionnaire. Reliability analysis indicated that the full-scale instrument had high internal consistency (Cronbach’s *α* 0.98). Factor analysis showed that the data on the questionnaire decomposed into three factors, which accounted for 72.6 % of the total variance: professionalism, collaboration, and communication. The generalizability coefficients (Ep^2^) were 0.76 for the surveys. Out of the 30 candidates, 26 participated in the knowledge test. The total mean score of the knowledge exam was 34.52, with scores ranging from 17 to 54.

**Conclusions:**

Based on this study’s results, we conclude that the instruments and procedures used have high reliability, validity, and feasibility in assessing the emergency physician in the emergency department in our clinical setting in the Middle East. The item analyses, reliability, and factor analyses all indicate that these instruments are effective in assessing emergency physicians.

## Background

Physicians in emergency care settings are especially obliged to carry out periodic evaluations due to their work with patients in critical conditions. Assessments such as multisource feedback are a crucial component for physician self-improvement. Such evaluations provide physicians with non-biased evaluations of both their strengths and their weaknesses, offering an opportunity for improvement in the fast-paced nature of their work.

Multisource feedback (MSF) is an evaluation tool whereby surveys assessing a variety of areas for physicians are administered among medical peers and colleagues [1]. Studies have shown that MSF results in an improvement in the communication skills of doctors with both patients and colleagues, as well as an overall improvement in medical care [2]. MSF is as reliable as objective methods as long as enough evaluators participate such that the bias factor is eliminated [3], although some argue that MSF is a subjective process [4]. Significant individual developments in family physicians’ medical care were found in a longitudinal study conducted over a 5-year period using MSF as the evaluation tool [5]. However, it is difficult to prove that this correlation is specifically as a result of the feedback physicians received from the MSF [2].

MSF has been particularly shown to be effective for emergency care physicians, based on a framework developed by the Council of Emergency Medicine Residency Directors in 2002 [6]. This framework focused on a variety of domains, including patient care, medical knowledge, practice-based learning, improvement, interpersonal skills, communication skills, professionalism, and system-based care [7]. A study conducted in Alberta, Canada, examined the MSF process as it applied to emergency physicians. Researchers developed a modified tool using input from emergency physicians who used the existing framework provided by CanMEDS and previously established tools. The study found MSF to be a useful evaluation tool for emergency care physicians that was both valid and reliable. The results found by this group of researchers were similar to those found by other studies examining MSF using a regulatory authority framework for other areas of practice [8].

Garra et al. conducted a multisource feedback assessment for emergency care physicians in their third year of residency using a modified humanism scale. They found that MSF was both a reliable and feasible tool to evaluate emergency care physicians and that it was also time-efficient [9].

We developed our own MSF tool to assess the emergency care physicians at our teaching hospital. The goal of this study therefore was to assess the emergency care physicians by using the MSF process and to assess the feasibility, reliability, and validity of the MSF process for the emergency physicians specifically and in the Middle East culture generally.

## Methods

### Study settings and participant

The study included all of the emergency physicians at a military teaching hospital in the Kingdom of Bahrain. The participants included 16 male and 14 female emergency physicians, for a total of 30 individuals. This sample size was determined by the total number of emergency physicians we have at our institution. The hospital consists of 450 beds, 322 physicians and dentists, and 1072 nurses and practical nurses. Yearly admissions include 21,462 inpatients and more than 347,025 outpatients. Our emergency department has the capacity for 48 beds and in 2013 saw 72,521 visits. Emergency calls responded to in that same year were 8872. The emergency department is currently using 3 resuscitation beds, 6 monitor beds, 2 gynecological beds, 1 isolation bed, 7 pediatric beds, and 11 short-stay beds.

### Raters

Past research examining the relationships between individuals and their evaluators has illustrated that the strength of the personal relationship has little to no effect on the outcome of the assessment, even when individuals selected their own assessors. Regardless of those findings, however, we hypothesized that the nature of the Middle Eastern culture may render those results inapplicable for our research study setting. As such, in our study, raters were assigned randomly to each emergency physician by an independent administrative team from the Training and Research department assignment, the only condition being that the physician has worked with their rater for a minimum period of 2 months. Each emergency physician was evaluated by three groups of raters: four fellow emergency physicians, four referral physicians from other departments, and four coworkers (nurses) from the emergency department itself. The survey instruments were distributed and collected in closed envelopes by an independent administrative team from the Training and Research department specifically formed for this task.

The introduction of the MSF in our organization encountered certain concerns and resistance from the emergency physicians at our institution in regard to their participation in this study. The physicians refrained to sign the consent or participate due to concern of such evaluation being used against them during their promotions and contract renewal.

Moreover, the idea of physicians being evaluated by nurses is new and not widely accepted in our culture. Hence, we opted to change our strategy to continue our study.

Eventually, two options were accessible to proceed with our study: first, to obtain an order or approval from the commander of the hospital of mandatory participation in the research; second, as described by some researchers, to implement the MSF process for the evaluation, we should first achieve the readiness of the organization and its employee. We finally agreed to continue with the second option.

Due to the different working shifts of the emergency physicians, three lectures (PowerPoint presentation) were conducted at different times to explain MSF and its functions while further assuring the physicians through an official letter by the Chief of Medical Staff stating that the evaluation would be solely used for improvement and formative feedbacks. Previous studies were used as a source to convince the physicians about the importance of involving nurses in this evaluation.

Furthermore, the physicians were informed that on success of this study in various departments, it might be further used as replacement for the yearly appraisal, which otherwise is solely the concern of the Head of the Department.

Although most were satisfied with the above idea, a one-on-one meeting was arranged for small groups who doubted the evaluation process and were unwilling to accept the idea.

After conducting the three lectures, a descriptive letter was given to the physicians by the Research and Research Ethic Committee with instruction on implementation of the MSF along with a description of the purpose of the study.

Through this letter, further clarification was made on the purpose of the study that it was exclusively intended for use in improvement of physician performance and we emphasized that the main purpose of this study is to assess the feasibility, reliability, and validity of implementing the MSF system in our hospital.

Furthermore, we explained that formative feedback will be provided to each emergency physician individually to provide feedback on potential areas for improvement.

The second challenge encountered based on our study in the Middle East culture was the response rates to the electronic questionnaire. The Middle East peoples’ acceptance and response to paper-based questionnaires is still higher than that of the electronic questionnaire.

To begin with, an electronic-based questionnaire was used and only 16 responses were received over a period of 2 weeks and two reminders. Based on this response rate, we concluded that the electronic-based questionnaire might not be feasible in our setting. Hence, we decided to change the strategy by using printed paper-based packets.

Reminder emails were sent as initial reminder and calls from the administrative team were made after 2 weeks of distribution of the printed paper-based packets surveys.

Following these criteria, 100 % response rates and collaboration were achieved from most of the raters.

For the survey-based evaluation to assess professionalism, communication skills, and collaboration, the cutoff score for the rated domains was set to 4.00 (out of a total of 5) based on the 25th percentile. Whereas, the candidates who score above 4.46 (out of a total of 5) based on the 75th percentile will be considered the best candidates.

### Instrument

We developed an instrument called the Bahrain Defense Force (BDF) instrument to assess physicians’ professionalism, communication, and collaboration modified from the physician achievement review instrument PAR which was used to assess physicians in Alberta [10]. We focused in our instrument to assess professionalism, communication skills, and collaboration only. To achieve face and content validity, a table of specification was constructed and a working group was involved in constructing the instrument. Expert opinion was considered as well. The instrument consisted of 39 items with 15 items to assess professionalism, 13 items to assess communication skills, and 11 items to assess collaboration. The instrument was constructed in a way that can be used by different groups of people such medical colleague emergency physicians, medical colleagues who are considered referral physicians from different departments, and coworkers from the emergency department.

The items on the instrument had a five-point response scale in the form of 1 = among the worst, 2 = bottom half, 3 = average, 4 = top half, and 5 = among the “best” with an option of “unable to assess” (UA). The self-instrument was a literal translation of the same instrument to the first person. After the committee had developed the questionnaires, they were sent to every physician whose work fit the profile for episodic care for feedback. Questionnaires were modified following that feedback.

### Statistical analysis

Each research question underwent a number of statistical analyses. Feasibility of the questionnaire was analyzed via response rates, average time required to complete it, and the low number of raters required to produce reliable results.

For each question on the surveys, the percentage, mean, and standard deviation of individuals who responded “unable to assess” were used to assess the validity of both the questions and the overall scores. Items in which more than 20 % of responders selected “unable to assess” may require either revision or removal altogether according to past findings [11].

We used exploratory factor analysis to determine which items on each survey belonged together (i.e., becoming a factor or scale). In this study, using individual-physician data as the unit of analysis for the survey, the items were intercorrelated using Pearson product moment correlations. The correlation matrix was then decomposed into principal components, and these were subsequently rotated to the normalized varimax criterion. The cutoff point for retaining items via varimax rotation was 0.40. Items were considered to be part of a factor if their primary loading was on that factor. The number of factors to be extracted was based on the Kaiser rule (i.e., eigenvalues 1.0) [12].

The factors or scales established through exploratory factor analysis were used to establish the key domains (e.g., professionalism) for improvement, whereas the items within each factor provided more precise information about specific behaviors (e.g., maintains confidentiality of patients, recognizes boundaries when dealing with other physicians, shows professional and ethical behavior). Physicians might then use their respective scores to improve their performance.

Using this type of analysis, we were subsequently able to examine whether we had reached our goal of aligning the survey items into the appropriate factors.

Cronbach’s coefficient was calculated for each scale and each factor, allowing us to analyze internal consistency reliability and stability of the instrument [13].

We then conducted a generalizability analysis to determine the Ep^2^. This allowed us to ensure that we had used enough raters as well as enough survey items in order to provide reliable data for each physician involved. We used an Ep^2^ of 0.70 to determine stable data. An Ep^2^ lower than 0.70 suggests low stability and would require either an increase in the number of raters or an increase in the number of items.

We used the following nested-design formula to determine Ep^2^ [14]:

$$ {\mathrm{Ep}}^2=\frac{\mathrm{Physician}\;\left(\mathrm{v}\mathrm{a}\mathrm{r}\;\mathrm{comp}\right)}{\mathrm{Physician}\;\left(\mathrm{v}\mathrm{a}\mathrm{r}\;\mathrm{comp}\right)+\mathrm{Error}\;\left(\mathrm{v}\mathrm{a}\mathrm{r}\;\mathrm{comp}\right)} $$

Although this type of design does not allow for estimation of the interaction effect of raters with physicians, it does allow for determination of the generalizability coefficient of raters.

#### Multiple choice questions (MCQs)

Although the MSF process appears to assess no cognitive skills, this approach is not ideal to assess knowledge and skills; these may be more accurately measured using MCQs, procedure-based assessments (PBAs), and objective structured clinical exams (OSCEs). To overcome this limitation, participating emergency physicians were assessed by a total of 70 MCQs and short-answer questions (SAQs). Out of a total score of 70 on the knowledge-based exam, success rate was set at 35 (50 %), whereby all those who scored below 35 were considered at high risk. The MCQs were constructed by experts in the respective fields tested. Face and content validity was measured by blueprint and a table of specification. The Kuder-Richardson formula 20 (KR20) was used to assess the reliability of the MCQs.

### Ethical approval

The research was approved by the research ethics committee at the BDF hospital. Written consent was obtained from the emergency physicians and verbal consent was obtained from the evaluators. This study was conducted from March 2014 to July 2014.

## Results

All of the emergency physicians in our hospital, a total of 30, including 16 males and 14 females, were assessed. Each emergency physician was assessed by a member of one of three different groups of clinicians. The first group included a medical colleague emergency physician, the second group comprised of a referral physician from another department, and the third group represented a coworker from the emergency department itself.

The total collected evaluation forms were 269, including 107 surveys from coworkers, 89 surveys from medical colleague emergency physicians, and 73 surveys from medical colleagues (referral physicians).

The total mean response rates were 74.2 %, and the self-reported average time needed to fill out each survey was 4.3 min, indicating a good feasibility of the questionnaire. Most of the questions were answered by the respondents. There were no single questions that exceeded the minimum 20 % of the “unable to assess” response by the raters which indicates revision or deletion of the items in the future is not required.

The whole instrument was found to be suitable for factor analysis (KMO = 0.967; Bartlett test significant, *p* < 0.05). Factor analysis showed that the data on the questionnaire decomposed into three factors which counted for 76.4 % of the total variance: professionalism, collaboration, and communication. Items were considered to be part of a factor if their primary loading was on that factor. The number of factors to be extracted was based on the extraction criteria of eigenvalues greater than 1.0 from the Kaiser-Guttman rule, the result which was subsequently triangulated by a priori specifying the number of factors to be extracted as 3. The descriptive statistics, item analysis, and factor analysis for each item are presented in Table 1.

**Table 1:** Descriptive statistics, item analysis and factor analysis

Q		N	M	SD	%UA	Self	SD	Factors identified by factor analysis		
								Comm	Colla	Profe
Q1	Maintains confidentiality of patients.	254	4.26	0.78	12.2	4.75	0.45			0.59
Q2	Recognizes boundaries when dealing with other physicians	258	4.24	0.81	11.3	4.76	0.44	0.54		
Q3	Recognizes boundaries when dealing with other healthcare professionals	259	4.18	0.91	10.6	4.60	0.60	0.63		
Q4	Shows professional and ethical behavior	261	4.28	0.85	10.3	4.70	0.47	0.79		
Q5	Is punctual and performs tasks in a time-appropriate manner	253	4.25	0.81	13.0	4.60	0.68			0.54
Q6	Is able to handle situations in a professional manner and exhibits self-control, avoiding emotional outbursts in stressful situations	260	4.13	0.89	10.6	4.45	0.68	0.67		
Q7	Respects patient’s autonomy and right to be involved in his/her own management	254	4.27	0.78	12.2	4.50	0.57	0.62		
Q8	Is reliable and responsible when performing his duties	260	4.23	0.94	10.6	4.70	0.56	0.66		
Q9	Is honest and handles his/her duties in a dignified manner	258	4.33	0.86	11.3	4.85	0.36	0.68		
Q10	Accepts constructive criticism and develops goals for improvement	248	4.10	0.92	14.7	4.60	0.50	0.64		
Q11	Respects cultural, individual, and role differences including age, gender, race, religion, disability, language, sexual orientation, and socioeconomic status.	254	4.28	0.85	12.2	4.80	0.36	0.70		
Q12	Follows institutional policies and procedures	255	4.28	0.87	12.3	4.55	0.61			0.63
Q13	Arrives on time to scheduled appointments and hospital activities	243	4.41	0.70	16.4	4.65	0.67			0.62
Q14	Manages healthcare resources efficiently	255	4.29	0.74	12.3	4.45	0.68			0.65
Q15	Leads with respect and fair treatment of colleagues	259	4.26	0.95	10.6	4.80	0.41	0.57		
Q16	Communicates efficiently and in a clear, understandable fashion with colleagues within his/her team.	261	4.32	0.80	10.3	4.75	0.44	0.63		
Q17	Communicates efficiently and in a clear, understandable, and compassionate way with patients.	256	4.29	0.78	12.0	4.55	0.51	0.59		
Q18	Allows the patient to elaborate about his condition	253	4.30	0.75	13.0	4.60	0.50			0.71
Q19	Communicates efficiently and in a clear, understandable, and compassionate way with patient’s families	257	4.29	0.77	11.6	4.55	0.60			0.67
Q20	Communicates clearly and effectively with other healthcare workers, e.g., nurses	257	4.28	0.83	11.6	4.70	0.47	0.66		
Q21	Explains what is being done for the patient during examination or procedures	243	4.28	0.82	16.4	4.70	0.57			0.77
Q22	Communicates purpose and results of investigations to patients well	250	4.25	0.77	14.1	4.65	0.58			0.76
Q23	Follows up appropriately and in a timely manner on patients’ hospital course	254	4.16	0.79	12.2	4.57	0.50			0.66
Q24	Communicates management options to patients in a clear, understandable way, taking into account the patients’ opinion	247	4.29	0.75	15.1	4.65	0.49			0.69
Q25	Displays empathy in dealing with patients by eye contact and verbal responses	249	4.20	0.87	14.4	4.70	0.57	0.52		
Q26	Summarizes the information given for the patient in small quantities, with concrete explanations, and understandable language	252	4.23	0.82	13.4	4.72	0.47	0.59		
Q27	Maintains calm in emergency situations, in order to communicate information clearly to his/her seniors	258	4.19	0.91	11.3	4.50	0.60	0.61		
Q28	Communicates accurate patient information to physicians from other departments when required to do so	259	3.35	0.75	10.6	4.70	0.47	0.58		
Q29	Manages to work well as part of a healthcare team	262	4.29	0.80	9.90	4.79	0.42		0.56	
Q30	Facilitates the learning of medical colleagues and coworkers	240	4.15	0.83	17.1	4.45	0.76		0.68	
Q31	Collaborates well with nurses and other healthcare workers	259	4.19	0.94	10.6	4.15	1.21		0.69	
Q32	Concerned about the safety of patients and coworkers	254	4.36	0.81	12.2	4.51	1.14		0.75	
Q33	Coordinates patient care efficiently	262	4.25	0.89	0.99	4.42	1.18		0.73	
Q34	Collaborates with other healthcare workers in order to achieve optimal patient care	259	4.28	0.82	10.6	4.50	1.14		0.76	
Q35	Participates in a system of call in order to provide care for patients	239	4.22	0.84	17.8	4.38	1.23		0.74	
Q36	Provides appropriate guidance and help to team members on regular bases	248	4.14	0.94	14.7	4.25	1.16		0.80	
Q37	Takes on extra work, when appropriate, to help the team	241	3.95	1.34	17.1	4.50	1.19		0.63	
Q38	Enables the team to achieve agreements for team process and collaborative completion of assignment	245	4.19	0.87	15.8	4.40	1.18		0.80	
Q39	Participates fully in collaborative process and fulfilled team agreements	236	4.16	0.91	18.9	4.58	0.98		0.78	

Reliability analysis (Cronbach’s *α* reliability of internal consistency) indicated that the full-scale instrument had high internal consistency (Cronbach’s *α* 0.98). The reliability for the factors (subscales) within the questionnaire had high internal consistency reliability (Cronbach’s *α* ≥ 0.91). G study analysis was conducted employing a single-facet, nested design. The generalizability coefficients (Ep^2^) were 0.76 for the surveys. The variance components and generalizability coefficients are presented in Table 2.

**Table 2:** Variance components and generalizability coefficients based on a D study

Number of rates	Ep^2^
4.000	0.622
5.000	0.672
6.000	0.711
7.000	0.742
8.000	0.767
9.000	0.787
10.000	0.804

Out of the 30 candidates, 26 participated in the knowledge test. The total mean score of the knowledge exam was 34.52, with scores ranging from 17 to 54. There were four doctors who scored below 35 and were considered at risk. Another four doctors did not appear for the exam for different reasons. The reliability analysis using KR20 for the internal consistency of the MCQs was measured and showed KR20 = 0.861.

In the non-cognitive domains (professionalism, communication skills, and collaboration), doctors 2, 12, 16, and 19 scored low below the 25th percentile and they were considered at risk. On the other hand, doctors 3, 7, 9, 10, 14, 18, 21, 22, 27, and 28 scored above the 75th percentile and they were considered the best candidates (Table 3).

**Table 3:** Number of observers and the mean score for knowledge, professionalism, communication skills, and collaboration for the emergency physicians

Doctor ID#	Proposed total number of observer	Actual number of observers	Knowledge/grades over 70	Total mean score	Mean score in professionalism	Mean score in communication	Mean score in collaboration
1.	12	7	54	4.74	4.76	4.78	4.66
2.	12	10	47	4.00	3.99	3.80	4.25
3.	12	9	46	4.48	4.47	4.55	4.43
4.	12	9	46	4.46	4.41	4.65	4.31
5.	12	11	45	4.40	4.37	4.40	4.44
6.	12	7	44	3.92	3.93	4.12	3.65
7.	12	9	43	4.80	4.79	4.80	4.81
8.	12	11	42	4.26	4.35	4.10	4.32
9.	12	7	42	4.51	4.56	4.53	4.40
10.	12	10	41	4.53	4.55	4.31	4.77
11.	12	8	40	4.26	4.21	4.30	4.27
12.	12	12	39	3.99	3.95	4.04	3.97
13.	12	7	39	4.19	4.24	4.23	4.08
14.	12	7	38	4.77	4.79	4.82	4.68
15.	12	8	37	4.25	4.20	4.27	4.29
16.	12	10	37	3.91	3.92	3.86	3.97
17.	12	8	37	4.17	4.08	4.33	4.18
18.	12	7	36	4.48	4.61	4.62	4.13
19.	12	10	36	3.96	3.93	4.02	3.95
20.	12	9	36	4.28	4.24	4.20	4.25
21.	12	10	35	4.61	4.57	4.56	4.72
22.	12	10	35	4.66	4.77	4.72	4.43
23.	12	9	33	4.12	4.28	4.10	3.97
24.	12	12	33	4.03	4.18	3.75	4.00
25.	12	10	32	4.45	4.35	4.56	4.46
26.	12	11	27	4.12	4.20	4.06	4.07
27.	12	7	–	4.61	4.61	4.55	4.68
28.	12	8	–	4.57	4.59	4.57	4.53
29.	12	8	–	4.45	4.40	4.60	4.32
30.	12	3	–	4.25	4.30	4.19	4.26

First quartile (25th percentile) = 4.00, second quartile (50th percentile) = 4.19, third quartile (75th percentile) = 4.46

When the results were later on presented in a meeting to the four consultants who were working in the emergency department, they were not surprised by the results; in fact, they confirmed that the results are very representative of each physicians based on their observation in the emergency department.

## Discussion

In this study, we evaluated the use and application of questionnaire-based assessments of the emergency physicians in our military teaching hospital. To our knowledge, this is the first study that investigates the feasibility, reliability, and validity of multisource feedback as a tool to assess emergency physicians in general and in the Middle East specifically.

In this study, we developed and evaluated a set of MSF questionnaires in order to conduct evaluations of our emergency physicians by fellow emergency physicians, by referral physicians from different departments, and by coworkers from within the emergency department. We were also aiming to assess the feasibility and reliability of these instruments and to begin to develop evidence for the validity of such assessments. Emergency physicians were assessed on a number of factors of practice that the regulatory authority and the physicians themselves believed to be important. The items and factors we used to develop our MSF overlap with some ACGME and CanMEDS competencies, although it had not been our original intention to assess those particular regulatory authority competencies [15]. The addition and retesting of new items and factors of our MSF questionnaire in the future would allow us to develop a new MSF that would in fact assess those sets of competencies, although some of those competencies may be tested by using different assessment methods.

Through this study, we reached our original goals and found that multisource feedback is a feasible and applicable type of evaluation tool in our Middle Eastern clinical setting, as evidenced by our adequate response rates. Although our strong response rates may be in part due to the newness of this type of study in our setting, these rates are consistent with the response rates for other similar types of studies. Through this study, we have gained initial evidence for the validity of our version of the MSF instruments, though establishing validity is a process that cannot be extrapolated from a single study. We found that nearly every item on the questionnaire could be answered by the raters, though there was a pattern that emerged for some questions that many of the respondents were unable to assess; these items must be reexamined for future studies. While some such items may be amenable to modification, others may need to be eliminated altogether.

Our exploratory factor analyses found that items did group together into factors in ways that are consistent with the intent of the table of specification. Regulatory authorities tend to be primarily concerned about the domains of professionalism and collaboration among emergency physicians [7]. As such, we focused our study around these particular factors, which we hope will provide the general direction for physician improvement, while the individual items will provide participating physicians with more specific feedback. Following the collection of the data, each physician received descriptive data (both means and standard deviations) on the scales as well as more specific individual items for himself or herself and for the group as a whole.

Future studies are required for the further examination of the validity of the instruments. For instance, it would be useful to determine whether emergency physicians who scored highly on this MSF assessment also perform relatively higher than their peers on other more objective assessments (e.g., accuracy of laboratory reports). Past research has been conducted to similarly assess the validity of criterion in different settings.

### Limitations

We recognize that this study had some limitations. Firstly, the study focused on emergency physicians in one hospital in the Kingdom of Bahrain, and as such, we cannot at this point extrapolate the data to apply it to different emergency physicians in other countries in the Middle East. Secondly, there were no patients used in this study. We tried to overcome this limitation by having one extra group of raters (referral physicians). Another limitation is our relatively small sample size, as the total number of our emergency physicians gave us only 30 participants. Though we used the maximum number possible given the capacity of our emergency department, had we had access to a larger number of participants, our results would have allowed us to examine patterns and draw comparisons between subgroups.

## Conclusions

We are faced with the challenge of assessing both the validity and reliability of assessing emergency physicians in their practice. We believe that our version of the MSF instrument for emergency physicians provides a feasible way of assessing physicians in our clinical setting and of providing guided feedback on various competencies and behaviors. Based on the results we have collected through this study, we believe our instruments and procedures have high reliability, validity, and feasibility. The item analyses, reliability, and factor analyses all indicate that these instruments are effective in assessing emergency physicians. . For further evidence of the validity of the multisource feedback instrument for emergency physicians, this study must be replicated in similar settings in the Middle East.
